# Bridging Microbiomes: Exploring Oral and Gut Microbiomes in Autoimmune Thyroid Diseases- New Insights and Therapeutic Frontiers

**DOI:** 10.1080/29933935.2025.2452471

**Published:** 2025-01-15

**Authors:** Daliya Abubakar, Hala Abdullahi, Ibrahim Ibrahim

**Affiliations:** a Research Department, Sidra Medicine, Doha, Qatar; b Women’s Department, Sidra Medicine, Weill Cornell Medical College-Qatar, Doha, Qatar

**Keywords:** Autoimmune thyroid diseases, gut microbiome, oral microbiome

## Abstract

Autoimmune thyroid diseases (AITDs) are the most common organ-specific autoimmune disorders characterized by thyroid dysfunction and immune system deficiencies. In recent decades, the role of the microbiome in autoimmune diseases has gained increasing attention, with emerging research linking gut microbiome alterations to the development of AITDs. This review summarizes current knowledge on the relationship between AITDs and the gut microbiome. Additionally, it emphasizes the role of the oral microbiome in AITDs, an area often overlooked in autoimmune research. Beyond the microbiome, the virome and mycobiome have been recognized as critical but underexplored components of the human microbiome, potentially contributing to immune dysregulation and the pathogenesis of AITDs. The review also explores modulating the microbiome for managing AITDs, including diet adjustment, the potential use of probiotics, postbiotics, symbiotics, and even fecal microbiota transplantation (FMT) to restore a balanced microbiome that may positively influence the immune system and, by extension, the course of AITDs. This review thoroughly explores the intricate relationship between AITDs, the gut, and oral microbiomes, paving the way for precision medicine applications in AITDs. Examining microbiota-thyroid interactions highlights the potential for targeted, personalized treatments and novel therapeutic therapies, guiding future therapeutic strategies for more effective and precisely tailored AITD management approaches.

## Introduction

The human microbiome has received increasing attention in recent years due to its fundamental contributions to a broad spectrum of physiological functions, including digestion, metabolism, immune system regulation, and even neurological health.^1^ Due to its profound effects on human biology beyond the genetic information found in human cells, it is often called a “second genome”.^1^ Different regions of the body host distinct microbiomes, each influenced by a range of factors such as diet, medications, and environmental exposure.^2^

The thyroid gland is an endocrine organ responsible for producing and secreting thyroid hormones and regulating iodine levels in the body. Approximately 90% of the thyroid’s output consists of the inactive hormone thyroxine (T4), while the remaining 10% is made up of the active hormone triiodothyronine (T3).^3^ Inactive thyroid hormone is converted peripherally to produce either activated thyroid hormone or another inert form. Thyroid-related disorders include autoimmune thyroid diseases (AITDs), in addition to others. However, this review seeks to highlight AITDs only. This review focuses on AITDs, while acknowledging that conditions like thyroid nodules, which can occur in the context of autoimmune diseases, are relevant but not the primary focus of this review. Thyroid cancer and other thyroid conditions unrelated to autoimmune disorders are excluded from this review.

This review emphasizes the current understanding of the various mechanisms implicated in the pathogenesis of AITDs and the relationship between AITDs and the human microbiome, including the gut and oral microbiome. It highlights the current strategies focused on targeting and modulating the microbiome in AITDs, which hold promise for developing future diagnostic tools and therapeutic interventions for these conditions.

## An overview of autoimmune thyroid disease

AITDs represent a group of organ-specific autoimmune disorders, including a broad spectrum of thyroid diseases, with Hashimoto’s thyroiditis (HT) and Grave’s disease (GD) being the most recognized forms.^3^ However, there are other immunogenic forms of destructive thyroiditis, such as postpartum thyroiditis and silent thyroiditis.^4^ HT is the leading cause of hypothyroidism, affecting between 5.8% and 14.2% of the general population, and is significantly more common in females, with a female-to-male ratio of 4:1.^5^ Interestingly, the incidence of hypothyroidism is 1.3% higher in countries with excess dietary iodine compared to regions where iodine intake is sufficient, indicating a link between iodine consumption and thyroid dysfunction.^6^ Conversely, GD is the primary cause of hyperthyroidism, accounting for 60% to 80% of hyperthyroid cases.^7^ Overall, AITDs manifest significant gender-based variations, with a higher incidence among women compared to men.^8^ Research shows that women are five to ten times more likely to develop AITDs than men, mainly due to genetic, hormonal, and immunological differences between the sexes.^9^

The emergence of a theory linking certain bacteria to autoimmune diseases, including thyroid gland autoimmunity, aligns with the growing understanding of the microbiome and its interplay with the immune system. The presence of microbes with epitopes resembling thyroid epitopes might lead to cross-reactive responses connected to the immune system.^10^ Previous studies have reported that *H. pylori* antigen epitopes resemble specific amino acid sequences in thyroid endogenous proteins, including segments of the thyrotropin receptor, thyroid autoantigens, and the sodium iodide symporter.^11^ Moreover, *Yersinia enterocolitica* bacteria activates early B cells that later mutate to target Thyroid-stimulating hormone receptors (TSHR). Mouse models showed that these antibodies cross-reacted with bacterial proteins.^12^ A recent study has shown that certain *Bifidobacteria* and *Lactobacillus* species share certain structural similarities with human thyroid peroxidase (TPO) and thyroglobulin (TG). This resemblance suggests that these bacteria may selectively bind TPO and TG antibodies via a “molecular mimicry mechanism,” potentially triggering AITDs^13^Although the precise mechanisms underlying these diseases remain unclear, a combination of immune dysregulation, genetic predisposition, and environmental factors is suggested.

**Figure 1.**
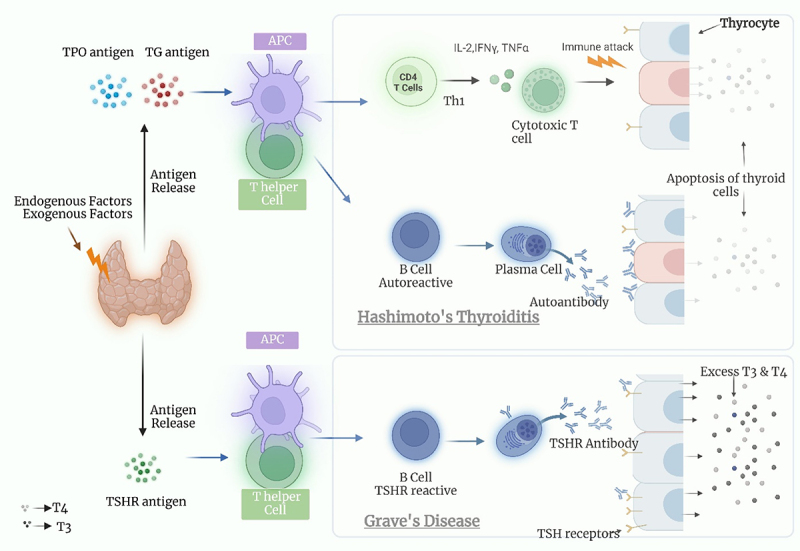
Pathogenesis of autoimmune thyroid diseases (Hashimoto’s thyroiditis and Grave’s disease). created with BioRender.

GD and HT are the most common AITDs that are often caused by immunological dysregulation. These conditions develop when the immune system accidentally targets the thyroid gland, resulting in thyroid gland inflammation and dysfunction.^3^ The primary distinguishing factor between HT and GD lies in the antibody profiles described in (Figure 1). Thyroglobulin antibodies (TG-Ab) and thyroid peroxidase antibodies (TPO-Ab) are higher in HT patients. Thyroid tissue is eventually destroyed as a result of the immune system attacking the thyroid gland and causing chronic inflammation. Anti-TPO and anti-TG-Ab are produced when lymphocytes, predominantly T-helper cells, invade the thyroid gland.^14^ At the same time, GD patients exhibit elevated thyroid-stimulating hormone receptor antibodies (TR-Ab). One of the key aspects of GD is the immune system attacking TSHR. Additionally, GD adopts the molecular mimicry mechanism through thyroid-stimulating immunoglobulins (TSI) mimicking the action of thyroid-stimulating hormone (TSH), leading to excess thyroid hormone production.^15^ Moreover, both diseases are characterized by the dysregulation of T-cell and B-cell responses, resulting in the generation of autoantibodies and inflammation.

In Hashimoto’s thyroiditis, the immune system produces antibodies like Thyroid Peroxidase Antibodies (TPO-Ab) and Thyroglobulin Antibodies (TG-Ab) that attack thyroid tissue antigens. These antibodies trigger inflammation in the thyroid gland by binding to these antigens, leading to chronic inflammation and damage to thyroid cells. This ongoing autoimmune process gradually destroys thyroid tissue, impairing its ability to produce sufficient thyroid hormones, ultimately causing hypothyroidism.

In Graves’ disease, the immune system produces thyroid-stimulating immunoglobulins (TSIs) that mimic thyroid-stimulating hormone (TSH). These TSIs bind to thyroid receptors, stimulating excessive production and release of thyroid hormones (T4 and T3), which, in turn, leads to hyperthyroidism.

**TPO**: Thyroid peroxidase, **TG**: Thyroglobulin, **APC**: Antigen-presenting cells, **TSHR**: thyroid-stimulating hormone receptor, **IL-2**: interleukin-2, **IFN-γ**: Interferon Gamma, **TNF-α**: Tumor Necrosis Factor Alpha, **T3: Triiodothyronine, T4**: Thyroxine.

Patients with GD, HT, and postpartum thyroiditis often display decreased CD8+ T cell counts in peripheral blood, a trend consistent with many autoimmune diseases. This reduction contributes to an increased CD4/CD8 ratio, which indicates a shift in immune balance. Additionally, these patients exhibit elevated levels of activated T cells expressing HLA-DR, a class II major histocompatibility complex (MHC) receptor encoded by the human leukocyte antigen (HLA) complex. The presence of HLA-DR is a marker of elevated immune activity. Within the thyroid tissue itself, T cell infiltrates consist of both CD4+ and CD8+ T cells, often in an activated state. However, in HT, there is frequently a predominance of CD4+ T cells, highlighting the key role of helper T cells in driving the autoimmune response and tissue damage in HT.^16^

AITDs are also characterized by the infiltration of B cells into the thyroid gland, where these intrathyroidal B cells actively produce antibodies. This emphasizes the thyroid as a significant source of autoantibodies in vivo, contributing to the autoimmune response. Additionally, B cells in other areas, such as the bone marrow and lymph nodes near the thyroid, also generate antibodies. The involvement of these cells outside the thyroid suggests a systemic component to the antibody production that drives the pathogenesis of AITDs, including both HT and GD.^16^

Regulatory T cells (Tregs) play a crucial, multifaceted role in AITDs. They primarily suppress autoreactive T cells, preventing them from attacking thyroid tissues. Tregs also regulate cytokine production by releasing anti-inflammatory cytokines like Interleukin-10 (IL-10) and transforming growth factor beta (TGF-β), which help maintain immune balance and reduce inflammation.^16^ Additionally, Tregs influence B cell responses, reducing the generation of autoantibodies that target thyroid antigens, which is a hallmark of AITDs.^17^ Tregs are also central to maintaining immune homeostasis and preventing autoimmune attacks on the thyroid gland. Their function extends beyond suppressing autoreactive T cells, as they also interact with dendritic cells, modulating antigen presentation and preventing excessive T cell activation, which is often seen in autoimmune conditions.^16,17^

Moreover, thyroid autoimmunity may be influenced by organ-specific and systemic autoimmunity, with 16.7% of GD patients reported to have another autoimmune disease. Thyroid autoimmunity has been linked to various conditions, including myasthenia gravis, vitiligo, chronic autoimmune gastritis, and rheumatoid arthritis.^18^

Recent research has emphasized the significant role of cytokines and chemokines in the development and progression of AITDs. One key factor is the infiltration of Th1 lymphocytes into thyroid tissue, which is driven by cytokine recruitment. These Th1 cells produce elevated levels of interferon-gamma (IFN-γ) and tumor necrosis factor-alpha (TNF-α), triggering a feedback loop involving the release of the Th1 chemokine CXCL10 from thyroid cells. CXCL10, first recognized as an IFN-γ-induced product, binds to chemokine receptor 3 (CXCR3) and plays a pivotal role in various autoimmune diseases, including GD, Graves’ ophthalmopathy, type 1 diabetes, systemic lupus erythematosus, and others. Elevated circulating levels of CXCL10 have been identified as markers of an aggressive inflammatory response in patients with HT, indicating its potential use as a biomarker for disease activity.^19,20^

### Genetic disposition

AITDs are significantly influenced by genetic factors, with immune-related genes contributing to individual susceptibility. Family and twin studies provide robust evidence supporting the genetic basis of AITDs,^21^ with research findings suggesting that nearly 80% of the risk for GD is hereditary.^22^ Several key genes involved in both immune regulation and thyroid function have been identified through linkage and association studies. These include thyroid-specific genes, such as TG and the TSHR, which are both critical to thyroid function.^23^ Additionally, immune-regulatory genes, like those found in the HLA locus, were among the first to be associated with both GD and HT. Specific HLA alleles are associated with either an increased or decreased risk of developing these autoimmune conditions, influencing how the immune system recognizes and targets thyroid tissue.^24^ Other important immune-related AITDs susceptibility genes include cluster of differentiation 40 (CD40), which is involved in immune system signaling, cytotoxic T lymphocyte-associated antigen 4 (CTLA-4), protein tyrosine phosphatase nonreceptor type 22 (PTPN22), and cluster of differentiation 25 (CD25), all of which play key roles in T cell activation and regulation.^25^ These genetic associations feature the complex interplay between immune regulation and thyroid function in the pathogenesis of AITDs.

### Environmental factors

AITDs can be triggered by various environmental factors. In GD, particularly, smoking is a significant risk factor, with both smoking and cessation posing risks. Cigarette smoke can impact iodine concentration in the thyroid and lactating breast.^26,27^ Iodine, essential for thyroid function, plays a pivotal role in thyroid dysfunction. Moderate iodine deficiency is associated with a lower prevalence of HT, whereas high intake increases the risk of developing HT.^28,29^ Moreover, infections can induce pro-inflammatory or regulatory immune responses.^30^ Whereas radiation exposure, whether therapeutic or environmental, is a well-established environmental factor linked to AITDs.^29,31,32^ Environmental toxins, such as polybrominated biphenyls and bisphenol A, are associated with thyroid cell toxicity and AITDs onset.^33–35^ Certain medications, such as interleukin-2 (IL-2), IFN-α, lithium, amiodarone, and antiretroviral therapy, are commonly linked to thyroid dysfunction.^31,36,37^ Various stress types have been linked to GD, but direct evidence linking stress to AITDs is currently lacking.^31,36,37^

### Other risk factors

AITDs, particularly HT, are more prevalent in women, suggesting a role for sex hormones and pregnancy-related immune changes. One hypothesis involves fetal micro-chimerism, where fetal cells that persist in the mother after pregnancy might trigger autoimmune reactions in the thyroid.^8,38^ The high prevalence of AITDs in females also underscores the potential influence of estrogens, which can modulate immune responses and may exacerbate autoimmune conditions. Estrogens enhance humoral immunity by increasing antibody production, which can contribute to the development of AITDs.^39^ Additionally, estrogen promotes the activation and survival of B cells, leading to higher antibody and autoantibody responses in females.^40^ In addition, the maternal immune system adapts to tolerate the semi-allogeneic fetus during pregnancy, and shifts in immune profiles, especially in the balance of pro-inflammatory and anti-inflammatory responses, are necessary for pregnancy success. However, the postpartum phase can trigger immune system reactivation, which is associated with the onset or exacerbation of autoimmune conditions such as AITDs.^41^

## Microbiome and autoimmune thyroid disease

### AITDs – gut axis

The human body harbors a diverse and dynamic community of microorganisms, collectively known as the microbiota. This includes a wide variety of bacteria, viruses, fungi, and protozoa that inhabit various parts of the body, such as the skin, mouth, intestines, and other mucosal surfaces.^42^ The role of the microbiota in autoimmune diseases has recently attracted considerable interest, with *Firmicutes* and *Bacteroidetes* being the dominant bacterial phyla involved.^16^ The relative proportions of these phyla are intricately linked to disease susceptibility, as shifts in their balance can reflect the overall health of the gastrointestinal ecosystem and provide insights into disease status. However, research into the *Firmicutes*/*Bacteroidetes* (F/B) ratio in AITDs has yielded conflicting results. Some studies, such as one conducted on GD patients, observed a higher F/B ratio in these patients compared to healthy controls,^43^ suggesting a possible link to inflammation associated with the disease. Conversely, other studies have found that the F/B ratio was significantly decreased in patients with AITDs,^44^ highlighting the variability in microbiome alterations depending on disease type and severity.

A recent meta-analysis revealed that patients with AITDs have reduced alpha (α) diversity and a lower abundance of certain beneficial microbiota than healthy controls.^45^ Alpha diversity reflects the complexity and richness of microbial communities. It is measured using indices such as Simpson’s and Shannon’s metrics, which account for both richness and evenness, while ACE and Chao1 are used specifically for estimating richness.^46^ These metrics provide valuable insights into the species abundance and distribution within a given environment, helping to assess microbial community structure. The same meta-analysis identified significant shifts in the gut microbiome composition of AITDs patients, particularly the substantial reduction of beneficial bacteria such as *Bifidobacterium* and *Lactobacillus* in AITDs patients compared to healthy controls.^45^ However, conflicting findings suggest that while these bacteria are generally considered beneficial for gut and immune health, their structural similarity to thyroid antigens, such as TPO and TG, raises the possibility of molecular mimicry, which may exacerbate autoimmune reactions.^13,47^ On the other hand, some studies argue that these bacteria may have protective roles by modulating immune responses and reducing inflammation, highlighting the complexity of their role in AITDs pathogenesis.^48^ Further research is needed to resolve these discrepancies and to determine whether these bacterial reduction in AITDs patients contribute to disease progression or results from it.

Conversely, pathogenic bacteria, such as *Bacteroides fragilis*, showed a significant increase in the AITDs group.^45^ Table 1. summarizes some of the microbial signatures associated with AITDs, highlighting the notable alterations in gut microbiota in these patients.

**Table 1.** Gut microbial signatures in AITDs.

Study	Associated AITDs	Country	Subject Details	Sequencing Methodology	Main Findings
^49^	HT	**China**	HT (*n* = 28), and healthy control (*n* = 16)	16S rRNA	Higher abundance of *Blautia, Roseburia, Ruminococcus_torques_group, Romboutsia, Dorea, Fusicatenibacter*, and *Eubacterium_hallii_group* genera, and lower abundance of *Fecalibacterium, Bacteroides, Prevotella_9*, and *Lachnoclostridium* in **HT patients** compared to healthy control.
^44^	AITDs	**Egypt**	AITDs (*n* = 20), and healthy controls (*n* = 30)	16S rRNA	Significant decrease in *Firmicutes*/Bacteroidetes ratio and lower abundance of *A. mucinophilia*, *Bifidobacterium*, *Lactobacillus*, and *F. prausnitzii* **AITDs patients** compared to healthy control. Positive correlation between *Bacteroidetes* and TR-Abs in **GD patients** and TPO-Ab in **HT patients**.
^50^	HT	**China**	HT (*n* = 29), and healthy control (*n* = 12)	16S rRNA	Higher abundance of *Escherichia-Shigella* and *Parasutterella*, and lower abundance in *Prevotellacea, Ruminococcaceae*, and *Veillonellaceae* in **HT patients** compared to healthy control.
^51^	HT GD	**Spain**	HT (*n* = 9), GD (*n* = 9), and healthy control (*n* = 11)	16S rRNA	Increase in gut bacterial richness in **HT** patients and a lower evenness in **GD patients**. Higher abundance of *Fusobacteriaceae*, *Fusobacterium*, and *Sutterella* in **GD patients** compared to healthy control. Higher abundance of *Streptococcaceae*, *Streptococcus* and *Rikenellaceae* in **HT Patients** compared to **GD patients**. Lower abundance of *Faecalibacterium* in **both AITDs** compared to healthy control.
^52^	GD	**China**	GD (*n* = 45), and health control (*n* = 59)	16S rRNA	Reduced alpha diversity in **GD** patients compared to healthy controls. Higher abundance of *Bacteroides* and *Lactobacillus* in **GD patients** compared to healthy controls. Subgroup analysis of **GD patients** revealed that *Lactobacillus* may play a key role in the pathogenesis of **AITDs** (significant correlations with specific thyroid function tests).
^53^	Pregnancy Hypothyroidism	**China**	Pregnant hypothyroidism (*n* = 30), and control (normal pregnant women, *n* = 30)	16S rRNA	Reduced beta diversity and higher abundance of *Prevotella* and *Paraprevotella* in **pregnant hypothyroidism** compared to control. Higher abundance of *Blautia* in controls.
^54^	GD	**China**	GD (*n* = 15), and health control (*n* = 14)	16SrDNA	Reduced alpha diversity and higher abundance of *Lactobacillus*, *Veillonella*, and *Streptococcus* in **GD** patients compared to healthy controls. Positive correlation between TR-Ab and the relative abundance of *Lactobacillus* and *Ruminococcus*.

Hashimoto’s Thyroiditis (HT), Autoimmune Thyroid Diseases (AITDs), Grave’s Disease (GD), Thyroid-Stimulating Hormone Receptor Antibodies (TR-Abs), and Thyroid Peroxidase Antibodies (TPO-Ab).

Over the past two decades, research has linked *Helicobacter pylori* (Hp) infection to AITDs. Hp may trigger HT by three key mechanisms: (1) CD4+ T cells recognize Hp epitopes that mimic thyroid H/K ATPase, causing Th1-mediated apoptosis; (2) dendritic cells present Hp antigens to naïve T cells, activating Th1 cells; and (3) INF-γ induces MHC II expression on thyroid cells, amplifying the immune response.^55,56^ Additionally, CagA-positive Hp strains share nucleotide similarities with TPO, raising the risk of AITDs.^57^

The gut microbiome affects thyroid hormone metabolism and bioavailability^58,59^ through several mechanisms, such as converting inactive thyroid hormone precursors into their active forms and modulating the sensitivity of thyroid hormone receptors.^60^ The gut microbiome also influences the bioavailability of levothyroxine and the enterohepatic cycle of thyroid hormones.^61^ The microbial imbalance, known as dysbiosis, can contribute to AITDs immunity dysfunction by disrupting the balance between pro-inflammatory and anti-inflammatory immune responses.^62^ The disturbed gut microbiome can compromise the integrity of the mucosal gut barrier, allowing harmful pathogens or antigens to translocate from the gut lumen into the systemic circulation. This breach can activate the immune system through various mechanisms, such as the stimulation of Toll-like receptor 4 (TLR4) by lipopolysaccharide (LPS), leading to the production of proinflammatory cytokines. These cytokines promote inflammation and may exacerbate conditions by over-activating the immune system, leading to tissue damage and chronic inflammatory responses.^63^ Furthermore, the gut microbiome is crucial for T-cell differentiation at barrier surfaces in both standard and pathogenic conditions. T-helper 17 (Th17) cells protect the host from harmful microbes, while Treg cells help regulate excessive effector T-cell responses. Gut dysbiosis can disrupt the balance between Treg and Th17 cells, potentially increasing susceptibility to inflammatory conditions.^60,64^ Additionally, gut dysbiosis can directly impact deiodinase activity, which is crucial in converting inactive thyroid hormones (T4) into their active form (T3), thereby influencing overall thyroid hormone levels and metabolism. The gut microbiome regulates thyroid hormones through its own deiodinase activity and by modulating TSH levels.^60^ Moreover, the gut microbiome is crucial for the absorption of essential minerals, such as selenium, zinc, and iodine, which are vital for the production and conversion of thyroid hormones.^60^ In cases where microbial imbalances affect gut function, the absorption of these minerals can be impaired, further compromising thyroid health and exacerbating AITDs. Short-chain fatty acids (SCFAs), particularly butyrate, are essential for immune regulation and gut health. Butyrate, produced by butyrate-producing bacteria within the *Firmicutes* phylum,^65^ strengthens the intestinal barrier and enhances mucosal immunity. A reduction in *Firmicutes* in in AITDs patients,^16^ may lead to increased intestinal permeability, or “leaky gut”, which subsequently results in lower butyrate levels. This decrease in butyrate is associated with higher levels of pro-inflammatory markers like interleukin-6 (IL-6) and TNF-α,^45^ indicating that altered gut microbiome may trigger immune responses that contribute to the onset or progression of AITDs.

Equally, maintaining balanced thyroid hormone levels is essential for gut health, as hormone imbalances can lead to gut dysbiosis. Impaired thyroid function disrupts several metabolic processes, including bile acid metabolism, which is essential for fat digestion and maintaining a healthy gut microbiome. In cases of hypothyroidism, reduced thyroid hormone production alters bile acid composition and circulation, potentially contributing to alterations in the gut microbiome. Moreover, thyroid hormones impact the gut microbiome through various mechanisms. Studies indicate that they influence gut barrier function, with hypothyroidism being linked to increased gut permeability and reduced gastroesophageal motility. These disruptions can further alter the microbiome composition, exacerbating conditions like dysbiosis and contributing to complications in both thyroid and gastrointestinal health.^66,67^ Furthermore, thyroid hormones play a role in host metabolism and immune modulation, adding complexity to the relationship between thyroid function and gut microbial communities.^68,69^ Research shows that shifts in thyroid function are associated with changes in the diversity and abundance of gut microbial species, reinforcing the bidirectional relationship between thyroid health and gut microbiota.^61^ The impact of gut microbial taxa on thyroid function can involve lower levels of beneficial *Firmicutes* and *Actinobacteria*, as well as shifts in the balance between *Firmicutes* and *Bacteroidetes*.^70^ Higher levels of pathogenic bacteria including *Yersinia enterocolitica*, *Helicobacter pylori*, *Borrelia burgdorferi*, *Clostridium botulinum* and *Rickettsia prowazeki* have been linked to thyroid health issues.^61^

**Figure 2.**
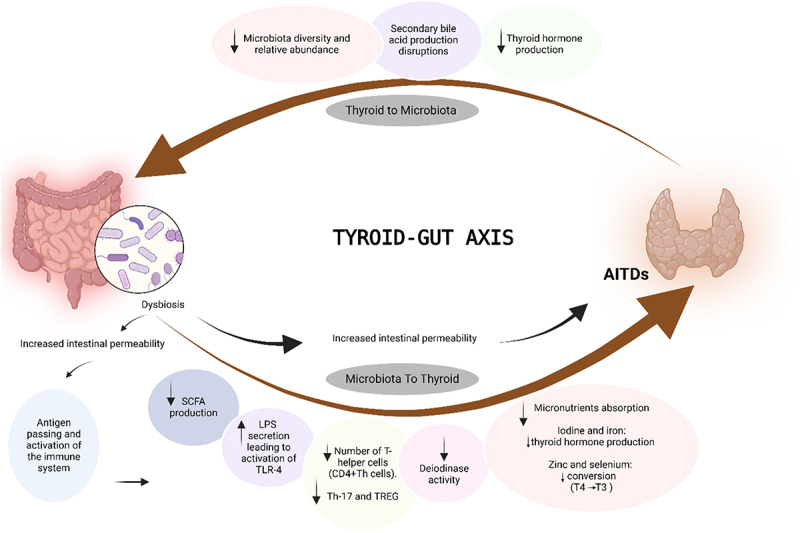
Pathways involved in the bidirectional communication between the gut microbiome and autoimmune thyroid disease pathogenesis. Created with BioRender.

The gut-thyroid axis plays a crucial role in maintaining endocrine balance and influencing AITDs, with studies suggesting a strong bidirectional relationship between AITDs and the gut microbiome. (Figure 2) provides a comprehensive overview of the gut-thyroid axis, illustrating the significance of this relationship.

The figure provides an in-depth exploration of the gut-thyroid axis, illustrating how dysbiosis of the gut microbiome can compromise the integrity of the mucosal gut barrier, allowing harmful pathogens or antigens to translocate into the systemic circulation. This translocation triggers immune activation through the LPS/TLR4 signaling pathway, leading to the production of proinflammatory cytokines. The gut microbiome also influences T-cell differentiation, affecting inflammatory responses. Moreover, changes in microbiome composition affect deiodinase activity, thus influencing thyroid hormone levels and metabolism. Additionally, impaired thyroid function, such as in hypothyroidism, can disrupt bile acid metabolism, potentially leading to dysbiosis. This bidirectional relationship underscores the importance of holistic approaches to thyroid health, considering both metabolic and microbial balance.

**SCAF**: Short Chain Fatty Acid, **LPS**: Lipopolysaccharide, **TLR-4**: Toll-Like Receptor 4, **TREG**: T-regulatory cells, **T3: Triiodothyronine, T4**: Thyroxine.

### AITDs- oral axis

In addition to the extensively studied gut microbiome, the oral cavity is the second largest and diverse microbiota after the gut, including bacteria, fungi, viruses, and protozoa, among others.^71^ The oral cavity is commonly inhabited by Gram-negative bacterial genera such as *Prevotella, Treponema*, *Bacteroides*, *Porphyromonas*, *Peptostreptococcus*, *Capnocytophaga*, *Eikenella,*, and *Fusobacterium*, *Actinobacillus*.^72^ Recent research has revealed that the oral cavity hosts more than 770 opportunistic, commensal, and pathogenic bacterial species.^73^

Emerging research suggests a potential bidirectional relationship between the oral microbiome and thyroid-related metabolic processes. Alterations in thyroid function may influence changes in the oral microbiome, and vice versa. However, further studies are needed to elucidate the nature and extent of these interactions.^74,75^ While the connection between thyroid function and the oral microbiome has been increasingly explored in recent years, the available data remains limited. This review aims to summarize the existing research on the thyroid-oral axis, providing a theoretical foundation for further mechanistic studies and offering a novel perspective on the development of microecological treatment strategies for thyroid disorders.

**Figure 3.**
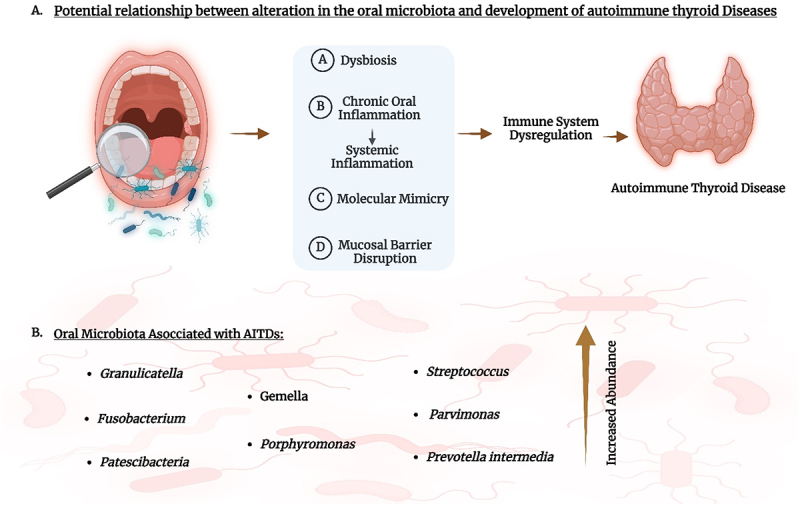
A. Potential relationship between alteration in the oral microbiome and development of autoimmune thyroid diseases. B. Oral microbiome species associated with AITDs.

The connection between the oral microbiome and the development of AITDs might be attributed to several plausible mechanisms (Figure 3). Immune system dysregulation is often linked to oral dysbiosis; alteration in the oral microbiome could lead to an inappropriate immune response, ultimately contributing to autoimmune disease.^76^ Additionally, chronic oral inflammation may give rise to a systemic inflammatory state that affects the thyroid as well as other organs.^77^ Immune system dysregulation is often linked to oral dysbiosis, with specific periodontal diseases such as gingivitis and periodontitis playing significant roles. Periodontal diseases occur due to the overgrowth of pathogenic bacteria like *Porphyromonas gingivalis*, *Tannerella forsythia* and *Treponema denticola*,^78^ which disrupt the balance of the oral microbiome. These bacteria can trigger an immune response by producing virulent factors that activate pro-inflammatory cytokines and immune cells.^78^ This activation leads to chronic local inflammation that, if persistent, can enter systemic circulation, promoting systemic inflammation and contributing to immune dysregulation.^79^ Emerging evidence suggests a complex relationship between thyroid disease, particularly hypothyroidism and periodontitis. Some conflicting studies report a higher prevalence of thyroid disease among individuals with good oral hygiene. Genetic factors, oral microbiome changes, and inflammatory cytokines may influence this relationship. Non-surgical periodontal therapies show promise in reducing inflammation and stabilizing TSH levels in hypothyroid patients. Limited studies suggest thyroxine may worsen periodontal health, highlighting the need for further research on thyroid medications’ effects on periodontal conditions.^80^ Moreover, Patients with hypothyroidism tend to have a higher prevalence and greater severity of periodontal disease symptoms compared to control groups.^81–83^

Created with BioRender.

Immune system dysregulation often correlates with oral dysbiosis, where alterations in the oral microbiome can provoke an inappropriate immune response, contributing to autoimmune diseases. Chronic oral inflammation can induce a systemic inflammatory state, impacting the thyroid and other organs and potentially exacerbating autoimmune thyroid conditions. Molecular mimicry is another mechanism linking oral microbiome to AITDs, where similarities in protein sequences between thyroid antigens and oral cavity microorganisms may activate autoreactive T cells, triggering autoimmune attacks against the thyroid. Moreover, changes in the oral microbiome may disrupt mucosal barriers, facilitating the passage of bacteria or their byproducts into the bloodstream, thereby instigating systemic effects and potentially autoimmune reactions in the thyroid or elsewhere in the body.

Molecular mimicry signifies another mechanism via which the oral microbiome is linked to AITDs. Thyroid antigens, as well as certain oral cavity microorganisms, may have analogous protein sequences. In response to this molecular mimicry, autoreactive T cells may become activated and potentially trigger an autoimmune attack against the thyroid gland.^84,85^ Furthermore, changes in the oral microbiome may potentially trigger autoimmune reactions in the thyroid or other parts of the body through a mechanism known as barrier disruption. Mucosal barriers, including the oral cavity, can become disrupted, allowing bacteria or their byproducts to pass into the bloodstream, causing systemic effects.^86^

Another theory proposes that changes in the oral microbiota can influence hormone regulation and metabolic processes. Certain oral bacteria, such as *Porphyromonas gingivalis*, *Prevotella intermedia*, and *Fusobacterium nucleatum*, are capable of producing metabolites and inflammatory mediators that can enter the bloodstream. These bacterial byproducts can potentially influence metabolic regulation by triggering systemic inflammation and immune responses. This inflammation can disrupt normal metabolic pathways, leading to conditions such as insulin resistance and altered glucose metabolism.^87^ One study observed that the phylum *Actinobacteria* was significantly less abundant in individuals with type 2 diabetes compared to controls, suggesting a potential association with the condition. Similarly, the presence of *Gemella* was reported to correlate with an increased risk of type 2 diabetes.^88^ While these findings highlight intriguing microbiome differences, they are based on cross-sectional analyses, and causality remains to be established.

Oral microbiota dysbiosis has been further linked with several autoimmune diseases, including inflammatory bowel disease, rheumatoid arthritis, systemic lupus erythematosus, Sjogren’s syndrome, ankylosing spondylitis, multiple sclerosis, autoimmune liver diseases, immunoglobulin A nephropathy, as reviewed by Huang et al., 2023.^89^

Ongoing research is increasingly focused on exploring the connection between the oral microbiome and AITDs. A recent study found a significant correlation between the presence of certain oral bacteria, including *Porphyromonas*, *Parvimonas*, and *Streptococcus*, and abnormal TSH levels.^74^ Another study correlated *Gemella morbillorum* to acute suppurative thyroiditis case.^90^ Subclinical hypothyroidism (SCH), a condition characterized by mild persistent thyroid failure,^91^ has been associated with alterations in microbiota composition, though the specific link to oral microbiota remains largely unexplored. Previous clinical research highlighted the abundance of *Prevotella intermedia* in the oral microbiota of SCH patients. Additionally, in an SCH mouse model, oral application of *P. intermedia* led to significant changes in the oral microbiota, exacerbated thyroid damage, and downregulated thyroid functional genes. Additionally, *P. intermedia* contributed to metabolic disruptions, including impaired glucose tolerance, insulin sensitivity, and lipid metabolism, as evidenced by increased triglyceride content and inflammatory infiltration in adipose tissue. Mechanistically, *P. intermedia* heightened CD4+ T cell and Th1 cell activity in the cervical lymph nodes and thyroid, suggesting an immune-mediated pathway. These findings indicate that *P. intermedia* worsens SCH symptoms by inducing immune imbalance, offering new insights into the role of oral microbiota in SCH pathogenesis.^92^ Table 2. provides a comprehensive overview of all studies examining the relationship between the oral microbiome and AITDs.

**Table 2.** Oral microbial signatures in AITDs.

Study	Associated AITDs	Country	Subject Details	Sequencing Methodology	Main Findings
^9^	Hyperthyroidism	**United States**	Participants with measures of thyroid function and oral microbiome (*n* = 2943)	16S rRNA	Reduced alpha diversity associated with hyperthyroidism.
^74^	Serum TSH levels	**China**	TSH level > 4.2 mIU/L (*n* = 20), TSH level no more than 4.2 mIU/L (*n* = 20), and Case-control matching	16S rDNA	Increased oral microbiome diversity associated with higher TSH levels. A higher abundance of *Granulicatella* is associated with higher TSH levels.
^93^	Serum TSH levels	**China**	high-TSH group (*n* = 22) low-TSH group (*n* = 24)	16S rDNA	Higher abundance of *Fusobacterium* is associated with high TSH levels.
^94^	Euthyroid HT	**Turkey**	(HT) females (*n* = 60) and healthy controls (*n* = 18)	16S rRNA	Higher abundance of *Gemella, Patescibacteria, Enterococcus, and Bacillus* in HT patients compared to healthy controls.
^95^	Pregnancy Hypothyroidism	**China**	Pregnant hypothyroidism (*n* = 30), and normal pregnant women (*n* = 31)	16S rRNA	Higher abundances of *Gammaproteobacteria, Prevotella, Neisseria*, and *Pasteurellaceae* in pregnant Hypothyroidism. Higher abundances of *Firmicutes, Leptotrichiace*, and *Actinobacteria* in healthy controls.

Hashimoto’s Thyroiditis (HT), Thyroid Stimulating Hormone (TSH), and Thyroid Peroxidase Antibodies (TPO-Ab).

Despite ongoing research, there remains a critical need for animal-model and pre-clinical studies to determine whether changes in the gut-oral microbiome are caused by the disease or if these microbiome changes result from the disease. These studies may reveal AITDs etiology, gut-oral microbiome elements separation, disease monitoring techniques, and new targeted therapeutic approaches, paving the way for continuous disease monitoring and improved treatments including microbiome-based targeted therapy.

### Emerging insights: the virome and mycobiome in AITDs

In light of emerging research, it is becoming increasingly evident that components of the microbiome beyond bacteria, such as viruses (virome) and fungi (mycobiome), may play significant roles in the pathogenesis of AITDs. While our review primarily focuses on bacterial influences, acknowledging these additional microbial communities is essential for a comprehensive understanding of AITDs.

#### Virome in AITDs

The virome, encompassing the collection of viruses residing in the human body, has been increasingly linked to immune system modulation and the pathogenesis of autoimmune diseases, including AITDs.^96^ Recent investigations have reported a high prevalence of viral agents in thyroid tissues, with multiple viral species in thyroid glands. Enteroviruses (EVs) were the most frequently detected, followed by human herpesvirus 6 (HHV-6) and parvovirus B19 (B19), while Epstein-Barr virus (EBV) and human cytomegalovirus (HCMV) were detected in a few cases. Importantly, virus distribution did not differ significantly between AITDs cases and controls, leaving open questions about their roles as causal factors, cofactors, or incidental findings.^96^

Another study highlights the potential roles of hepatitis C virus (HCV) and B19 in AITDs, demonstrating the presence of B19 in thyroid tissues of patients with HT, GD, and thyroid cancer, though its pathogenetic role remains unclear. Chronic HCV infection was linked to elevated thyroid autoantibodies, SCH, and increased prevalence of AITDs and papillary thyroid cancer, particularly in those with HCV-associated mixed cryoglobulinemia. These findings highlight the importance of monitoring thyroid function and nodules in HCV patients with risk factors such as female gender, elevated TPO-Ab, and hypoechoic or small thyroid gland.^97^ A recent study further explored the role of EBV in thyroid dysfunction, particularly in GD patients. The findings demonstrated that EBV reactivation induces IgM-dominant thyrotropin-receptor antibodies (TR-Abs). While these antibodies did not interfere with TSH receptor binding or hormone production, they caused significant damage to thyroid follicular cells via complement activation.^98^ These studies highlight the potential role of the virome in the onset and progression of thyroid disorders, highlighting its significance within the broader framework of viral influences on thyroid autoimmunity.

#### Mycobiome in AITDs

The mycobiome, comprising fungal communities residing in the human body, has also been implicated in autoimmune conditions,^99^ though its role in AITDs is less explored. Fungal organisms, such as *Candida* species, are known to influence immune responses by producing antigens that can modulate inflammatory pathways.^100^ Overgrowth of *Candida* has been associated with increased intestinal permeability, facilitating the translocation of microbial antigens and potentially triggering autoimmune responses.^101^ Similarly, the mycobiome has been explored in other thyroid-related conditions, such as papillary thyroid carcinoma, where dysregulation of the mycobiome was observed.^102^

Although the bacterial microbiome has been the primary focus of research in AITDs, the virome and mycobiome present exciting opportunities for future investigation. Exploring the intricate interactions between these microbial communities and the host immune system could provide valuable insights into the underlying mechanisms driving the development and progression of AITDs. Continued research in these areas is essential to fully understand their roles and uncover potential therapeutic strategies.

## Therapeutic approach: modulating the microbiome

Studies on the connection between the microbiome and AITDs are currently an active area of research. Moreover, specific microbiome-based therapies targeting AITDs are in the early developmental stage.^103^ Various strategies are currently being explored to address AITDs, including the use of probiotics, prebiotics, and synbiotics to support gut health, dietary interventions, and fecal microbiota transplantation (FMT). These approaches aim to modulate the microbiome and enhance immune system balance, and ongoing research investigates their effectiveness.

### Probiotics, prebiotics, synbiotics, and postbiotics in AITDs management

The use of probiotics, prebiotics, synbiotics, and postbiotics offers promising therapeutic avenues for managing AITDs. **Probiotics** are live, beneficial bacteria that, when consumed in sufficient quantities, can successfully reach the colon and enhance the host’s overall health.^104^ Few studies have insinuated the role of probiotics in modulating immune responses and boosting a balanced gut microbiome, which, in turn, will impact thyroid-immune-related diseases. The potential anti-inflammatory and immune-modulating properties of probiotics, which underscore the significance of these probiotics in autoimmune disease management, have been extensively studied.^105^ *Lactobacillus reuteri* supplementation in mice resulted in an increase in the thyroid mass, free T4, and other physiological indicators, such as physical activity level.^106^ Another animal model study examined the impact of probiotics containing *Lactobacilli* and *Bifidobacteriaceae* on levothyroxine. The study revealed a notably reduced T4 adjustment requirement when compared to the control group. This finding is explained by the possibility that microbiome change enhances levothyroxine availability and stabilizes thyroid function. The study concluded that probiotics help reduce variations in blood hormone levels. In addition, probiotics may increase the availability of the bacterial enzymes ß-glucuronidases and sulfatases, which regulate the deconjugation of iodothyronines.^107^

**Prebiotics**, which are non-digestible fibers that promote the growth of beneficial gut bacteria, help regulate the immune system and improve gut health. Research has shown that prebiotics can aid in restoring microbial balance, enhancing SCFAs production such as butyrate. One study involving a combination of berberine, a compound with prebiotic effects, and methimazole in GD patients revealed improved thyroid function and better modulation of the gut microbiome, suggesting prebiotics’ potential in thyroid health. The introduction of the potential prebiotic berberine led to changes in the gut microbiota composition of patients with GD. It promoted the growth of the beneficial bacterium *Lactococcus lactis*, while reducing levels of pathogenic bacteria including *Chryseobacterium indologenes*,^108^ and *Escherichia coli*,^109,110^ thereby supporting the regulation of microecological balance in these patients.

**Synbiotic** supplementation, which combines probiotics and prebiotics, has shown promising results in pregnant patients with HT. A study demonstrated that supplementation using probiotics such as *Bifidobacterium*, *Lactobacillus acidophilus*, *Enterococcus faecalis*, and *Bacillus cereus*, alongside prebiotics like inulin and oat fiber, led to a significant reduction in TSH levels, while maintaining TSH within the normal range. This reduction also resulted in a decreased requirement for levothyroxine dosing. Additionally, participants reported improvements in symptoms like fatigue. The supplementation also increased free T3 (fT3) levels, highlighting its potential in supporting thyroid function in hypothyroidism patients.^111^

**Postbiotics** include any substance released or produced by microbes via metabolic activity that either directly or indirectly benefits the host. Postbiotics work through five different mechanisms: (1) altering the resident microbiome, (2) boosting the functions of the epithelial barrier, (3) altering the local and systemic immune responses, (4) altering the systemic metabolic responses, and (5) modulating systemic signaling through the nervous system.^112^ Purified microbial metabolites, such as butyrate, have been demonstrated to have some anti-inflammatory properties.^113^ Therefore, infusions of SCFA mixes can have a significant impact on thyroid function. Postbiotics may also indirectly modify the microbiome, for example, by transmitting quorum sensing and quorum quenching molecules^114^ or by transmitting lactic acid, which can be absorbed by some microbiome members, resulting in the production of SCFAs, including butyrate.^115^

### Role of micronutrients in AITDs management

The interaction between diet, microbiome, and autoimmunity is growing, especially in AITDs research. Diet strongly influences gut microbiota and immune function, and in genetically susceptible individuals, certain foods may trigger or worsen autoimmune responses, though the exact mechanisms are still unclear..^116^

Research indicates notable differences in dietary patterns between individuals with HT and those without the condition. A study found that HT patients reported higher consumption of animal-based foods, including meat, fish, and dairy products, compared to healthy controls. Conversely, the control group had a greater intake of plant-based foods such as legumes, fruits, and vegetables.^116^ These dietary habits may influence oxidative stress and inflammation, factors associated with HT. Another study observed that HT patients had higher dietary inflammatory index (DII) scores, indicating a more pro-inflammatory diet, and lower dietary total antioxidant capacity (DTAC) compared to healthy individuals. This suggests that diets rich in anti-inflammatory and antioxidant compounds may be linked to a reduced risk of developing HT.^117^ Triglycerides in animal fats are known to contain saturated fatty acids (SFA), which have been associated with the development and progression of various chronic diseases due to their role in promoting inflammatory responses.^118^ SFA can activate TLR4 and results in the production of proinflammatory cytokines, disturbance of cellular metabolism, and impairment of gut barrier function.^119^ Two recent studies found that a high-fat diet can lead to thyroid dysfunction in rats and contribute to hypothyroidism by lowering total free thyroxine (fT4) and T4 levels while increasing TSH levels.^120,121^ These findings were consistent with another study,^122^ which concluded that the group with higher intakes of animal fats had a considerably higher frequency of positive plasma TPO and/or TG antibodies. High consumption of SFA may result in an impaired gut-microbial population, which, in turn, may lead to systemic inflammation. At the same time, low intake of SFA is linked to increased α-diversity in the gut microbiome and an increased abundance of specialized bacteria that are capable of degrading dietary fibers.^123^

### Fecal microbiota transplantation (FMT) in AITDs

Numerous investigations have examined the potential application of FMT as a therapeutic strategy for autoimmune disorders. FMT involves the transfer of a donor’s stool sample, which includes a diverse mix of bacteria, viruses, fungi, metabolites, and other microbial components, with the goal of restoring the gut microbiota balance in the recipient.^124^ This procedure can be administered through various methods, such as colonoscopy, retention enemas, nasogastric and nasoduodenal tubes, upper endoscopy, FMT capsules, and rectal tubes.^125^

Several studies exploring the therapeutic potential of FMT for GD have shown promising results.^126^ FMT from primary hypothyroidism patients led to a gradual reduction in serum total thyroxine levels in mice, compared to those receiving FMT from healthy donors, with a significant decrease observed six weeks post-FMT.^127^ In an experimental murine model, FMT was used to explore the gut microbiome’s influence on thyroid hormones and metabolism. Hyperthyroid animal-model receiving FMT from control animals showed gut microbiota changes, a greater reduction in T3 and T4 levels, increased liver type 2 deiodinase expression, and improved recovery of resting metabolic rate. These findings suggest that gut microbiota alterations may reduce hyperthyroid-induced thermogenesis.^128^

FMT is generally safe but can have side effects such as diarrhea, abdominal cramps, nausea, and fever, which are usually mild and temporary.^129^ Serious complications, though rare, may include infections from improperly screened donors and risks associated with the procedure itself, such as infection or perforation. More severe side effects, include sepsis or exacerbation of underlying conditions.^129^

Recent studies indicated that FMT is not always effective, and that the success of the treatment relies heavily on factors related to both the donor and recipient,^130^ impacting microbiome engraftment and treatment efficacy. This highlights the need for careful donor selection and recipient preparation to improve outcomes.

### Engineered bacterial therapeutics in AITDs

Metabolic syndrome, which is group of conditions that increase the risk of cardiovascular diseases, along with insulin resistance and type II diabetes mellitus,^131^ has been linked to various thyroid dysfunctions, particularly HT.^132^ The use of engineered bacterial therapeutics has been employed in the treatment of metabolic syndrome. For instance; genetically modified *Escherichia coli Nissle 1917 (EcN*) is used to express genes that convert fructose-a common sugar in Western diets linked to metabolic disorders and cardiovascular disease-into mannitol, a prebiotic that has been shown to protect against metabolic syndrome,^133^ highlighting potential strategies to address both metabolic and thyroid-related dysfunctions.

### CRISPR-dCas9 gene regulation for microbiome modulation in AITDs

CRISPR-dCas9 (deactivated Cas9) variant, a modified form of the CRISPR-Cas9 gene-editing system,^134^ can regulate gene expression in the microbiome without changing the genomic sequence. This is achieved by utilizing a catalytically inactive Cas9 (dCas9) protein, which binds to target DNA sequences without cutting them. By attaching transcriptional repressors or activators to dCas9, researchers can modulate the expression of specific genes, allowing precise functional studies and targeted gene regulation.^135^

A study conducted in *Escherichia coli* utilized CRISPR-dCas9 for genome-wide screening, identifying key genes involved in phage infection and antibiotic resistance, demonstrating its potential in microbial functional genomics.^136^ Additionally, researchers developed a method to express dCas9 in bacteria based on their metabolic states, enabling selective gene regulation within complex microbiomes for targeted research and therapeutic applications.^137^

While CRISPR-dCas9 holds significant potential, its application in modulating the microbiome’s metabolic output offers promising avenues for improving AITDs outcomes. For instance, dCas9 could be used to upregulate genes responsible for the production of anti-inflammatory metabolites, such as SCFAs, which are known to influence immune regulation.^134^ Additionally, it could be employed to downregulate genes associated with the synthesis of pro-inflammatory metabolites, such as trimethylamine N-oxide (TMAO), which exacerbates autoimmune responses.^138^ By selectively modulating the production of metabolites that interact with host immune pathways, CRISPR-dCas9 may enable personalized therapeutic approaches to restore immune balance and reduce thyroid inflammation.

CRISPR-based technologies have the potential to selectively target and regulate specific microbial genes, offering novel strategies for manipulating microbiome composition and function.^134^ For instance, CRISPR-Cas9 could be used to downregulate virulence factors in pathogenic bacteria or enhance beneficial metabolite production in commensal species.^139^ These advancements provide promising tools for developing microbiome-based therapies in various disease contexts, including AITDs. Future research should focus on identifying specific microbial targets and optimizing delivery systems to harness this potential effectively.^140,141^

### Phage therapy: targeting microbiome dysbiosis in AITDs

Phage therapy, which uses bacterial viruses (bacteriophages) to infect and destroy bacterial cells specifically,^142^ is a novel approach to modify microbiome diversity. Phage therapy not only influences microbiome diversity but also impacts the abundance of specific bacterial species through targeted interactions. Specific phages infect specific bacteria by recognizing unique receptors on their surfaces, often resulting in the lysis and death of the host bacteria.^143,144^ This targeted reduction can create ecological niches that allow other bacterial populations to flourish, thereby reshaping the overall microbial community structure and altering microbiome diversity. For example, it can reduce harmful populations such as *Escherichia coli* while promoting the growth of beneficial bacteria like *Eubacterium* species, reshaping the overall microbial community structure.^145^

Additionally, phages modulate host-relevant metabolites by reshaping microbial communities. They increase amino acids like serine and threonine by promoting mucin-degrading bacteria (*Akkermansia muciniphila and Bacteroides vulgatus*) and alter bile salt metabolism by enhancing bile salt hydrolase (BSH) activity. These findings demonstrate phages’ potential to influence metabolite production with host impacts.^146^ These microbial and metabolite changes may influence systemic immune responses and inflammation, which are relevant to AITDs.^147^

Phage therapy offers a precise tool for managing microbiome-related dysbiosis, a hallmark of AITDs, by restoring microbial balance and reducing low-grade chronic inflammation linked to autoimmune conditions.^146,147^ However, further research is needed to understand its long-term efficacy, potential resistance mechanisms, and scalability in clinical settings. Nonetheless, these findings underscore phage therapy’s potential as a transformative approach to addressing dysbiosis and improving outcomes in AITDs.

**Figure 4.**
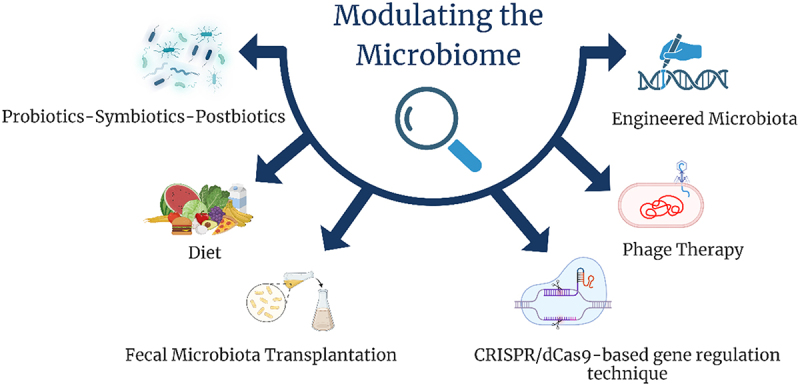
Modulating the microbiome. Created with BioRender.

There are limits to the existing pre-clinical and clinical findings on modulating the microbiome (Figure 4) for the treatment of metabolic and autoimmune diseases in general, and AITDs specifically. However, research on these areas is ongoing and could alter treatment in the future.

Current and emerging strategies aim to modulate the microbiome to treat autoimmune thyroid diseases. These approaches include probiotics, prebiotics, synbiotics, diet modulation, and innovative methods such as fecal microbiota transplantation (FMT). Potential future therapies may involve genetic modification of microbial populations using tools like CRISPR/dCas9-based gene regulation technique, Engineered microbiota, and phage therapy, offering targeted interventions without disrupting the overall microbiome.

## Conclusion

The connection between AITDs and the human microbiome underscores the intricate interplay between microbial ecosystems and thyroid health. The gut-thyroid axis highlights the bidirectional link between gut health and thyroid function, with the oral microbiome playing an important role in maintaining a healthy gut, in addition to influencing thyroid function via different mechanisms. Additionally, the virome and mycobiome are increasingly recognized as critical contributors to immune regulation and potential modulators of AITDs.

Promising therapeutic strategies, such as probiotics, synbiotics, postbiotics, FMT, phage therapy, and genetic modification of microbial populations, provide potential avenues for intervention. Personalized approaches, including tailored nutrition and micronutrient management, are also essential to addressing individual variations in microbial and viral profiles.

However, to advance our understanding of the microbiome, virome, and mycobiome in AITDs, there is a pressing need for robust animal models that replicate the complex interactions within the human thyroid-microbiome axis. Such models would enable in-depth exploration of underlying mechanisms and provide a controlled environment for testing therapeutic interventions. Future research should focus on integrative studies and animal model development to uncover these microbial ecosystems’ systemic impacts on AITDs. These efforts will pave the way for innovative diagnostic tools and therapeutic strategies, ultimately improving patient care and outcomes.
